# Antimicrobial management of dental infections: Updated review

**DOI:** 10.1097/MD.0000000000038630

**Published:** 2024-07-05

**Authors:** Faraj Mohammed Abdullah, Qais Y. Hatim, Amjad I. Oraibi, Thamir Hani Alsafar, Tahani Abdulaziz Alsandook, Wael Lutfi, Hany A. Al-Hussaniy

**Affiliations:** a Dean of Al-Manara College for Medical Sciences, Amarah, Iraq; b Al-Manara College for Medical Sciences, Amarah, Iraq; c Department of Dentistry, Alturath College, Baghdad, Iraq; d Head of Dentistry Department, Alturath College, Baghdad, Iraq; e Head of the Dentistry Department, Al-Manara College for Medical Sciences, Amarah, Iraq; f Department of Pharmacy, Bilad Alrafidain University College, Baqubah, Iraq; g Dr Hany Akeel Institute, Iraqi Medical Research Center, Baghdad, Iraq.

**Keywords:** antibiotic prophylaxis, antimicrobial resistance, dental caries, low-level laser therapy

## Abstract

Dental infections, which include anything from severe periodontal illnesses and abscess forms to routine tooth caries, are a major public health risk. This review article focuses on the pathophysiology and treatment of dental infections. A narrative review was conducted based on several published articles, relevant journals, and books in Google Scholar PubMed using the keywords dental caries, periodontal disease, gingivitis, and related diseases; we excluded duplicated information. Our review illustrated the types of dental infections and the proper antimicrobial drug that is suitable for this disease. Drawing from recent research findings and clinical evidence, we explore the spectrum of bacteria commonly associated with dental infections and their susceptibility profiles to various antibiotics. Emphasis is placed on understanding the mechanisms of antibiotic action and resistance in the context of dental pathogens, shedding light on optimal treatment regimens and potential challenges in clinical management. Additionally, we go over the clinical consequences of antibiotic therapy in dentistry, taking into account factors like patient selection, dose guidelines, and side effects. The management of dental infections through antimicrobial strategies has undergone significant advancements, as evidenced by this updated review. Besides the normal methods, emerging technologies such as 3D printing for drug delivery of antibiotics and disinfectants hold promise in enhancing treatment efficacy and patient outcomes. By leveraging the precision and customization afforded by 3D printing, dentistry can tailor antimicrobial interventions to individual patient needs, optimizing therapeutic outcomes while minimizing adverse effects.

## 1. Introduction

Dental caries, periodontal disorders, and pulpal necrosis can cause dental infections. These infections can seriously harm the oral cavity’s soft and complex structures. Pain, fever, and edema are frequent signs of dental diseases. The early treatment of infected teeth involves surgery and endodontics, followed by antibiotic medication.^[1]^

In dental practice, dentists prescribe antibiotics, especially during dental treatment and as a preventive measure. For instance, in patients with prosthetic joints, infectious endocarditis, metabolic disorders like diabetes,^[2]^ and other conditions, antibiotics are given to prevent acute attacks; they are also administered before tooth extraction.^[3]^ There are few conditions in dentistry where systemic antibiotics may be used since oral hygiene practices, including topical antiseptics, antibiotics, and surgical intervention,^[4]^ are the best ways to treat most dental and periodontal disorders.^[5]^ The use of antibiotics in dentistry is typified by the empirical use of a limited number of short-term, broad-spectrum antibiotics, such as Amoxicillin, metronidazole, or clindamycin.^[6]^ In dentistry, the main work is surgery and endodontic treatments; the dental work is the main issue, and antibiotics are sometimes indicated.^[7]^ Only around 12% of dentists were found to give antibiotics appropriately and accurately, demonstrating the need for thorough guidelines. Misuse and overuse of antibiotics in dentistry will lead to side effects, and the emergence of antimicrobial resistance (AMR) results in treatment failure. It may hurt a vast number of populations all over the world. The creation of prescription guidelines and educational programs to promote the responsible use of antibiotics and discourage their irrational use are necessary to prevent the onset of AMR.^[8]^ Despite continuous education about essential antibiotic guidelines, the World Health Organization reported that the emergence of resistance is increasing.^[9]^ For example, resistance against clindamycin is increased by 46.7% for Amoxicillin, 39.2%, doxycycline by 25%, and metronidazole by 21 7%, but the combination of Amoxicillin and metronidazole by 6.7% only. Misuse and overuse of antibiotics have led to the development of AMR in a wide range of microorganisms, which leads to the consequent inefficacy of commonly used antibiotics.^[10]^ Antibiotics are often used during dental operations to treat prophylactic, local, focal, odontogenic, and non-odontogenic infections.^[11]^ Odontogenic infections are the most frequent, affecting periodontal and dental structures such as dental caries, pericoronitis, periodontitis, pulpitis, or pulpal necrosis.^[12]^ non-odontogenic infections start in extra-dental structures such as mucous glands, tongue, paranasal sinus, and sialadenitis. They can cause severe infections that can migrate to deep backspaces and sometimes lead to serious complications and death.^[13]^

Specific dental procedures, such as extractions, surgical periodontal procedures, implant placement, re-implantation of teeth, endodontic procedures or surgeries, subgingival placement of antibiotic fibers or strips, and intraligamental local anesthetic injections, increase the risk of dental infection in susceptible patients. Antibiotic prophylaxis is required to avoid dental and extra-dental issues.^[14]^ Dental infections could be cured by surgical intervention; the earlier the surgical management of the infected tooth, the better clinical outcomes. In severe cases, surgical intervention includes debridement, irrigation, and incision and drainage. Furthermore, in patients with signs of systemic involvement, administration of intravenous antibiotics according to bacterial cultures and sensitivity is suggested.^[15]^ Present guidelines indicate that antibiotics should be prescribed 2 to 3 consecutive days after surgical treatment.^[16]^ The surgical cut in the soft tissue (incise) and drain the pus as the antibiotic is not working in the presence of pus (this is because pus contains antibiotic inhibitors). A root canal (RC) can help get rid of the source of infection; the dentist accesses the cavity in the teeth, removes the diseased and infected central tissue (pulp), disinfects the canal using an irrigation solution, applies antibiotic dress in the canal, and restoration of the access cavity.^[17]^

Suppose the affected teeth cannot be saved. In that case, the dentist will extract the tooth and prescribe antibiotics after extraction to prevent infection (in addition to antibiotics before extraction, particularly in immunosuppressed patients).

However, prescribing antibiotics is not recommended if the infection is limited to the abscessed area (localized). If the patient has no systematic manifestations but the disease has spread to nearby teeth or jaw, the dentist will likely prescribe antibiotics to stop the spreading of the infection. Dentists prescribe broad-spectrum antibiotics with bactericidal effects or 2 bactericidal antibiotics in immunosuppressed patients.

Low-level laser therapy^[18]^ and photodynamic therapy^[19]^ are 2 alternative antiseptic techniques. A treatment has been used to treat infected wounds to lessen bacterial growth and inflammation. On the other hand, photodynamic therapy has effectively treated localized infections, including burns, abscesses, wounds, and periodontal infections, as well as eliminated bacteria. Dental infections should be treated as soon as possible because they can cause severe and irreversible side effects like meningitis, thrombosis, orbital abscess, osteomyelitis, brain abscess, airway obstruction, carotid sinuses, septicemia, and loss of vision.^[20]^ It is advised to administer antibiotic prophylaxis before routine invasive dental care surgeries. Additionally, a new challenge for collaboration between dentistry and medical researchers has emerged from the link between oral infections and myocardial/cerebral infarction.

This review illustrates types of causative microorganisms, types of dental infections, and appropriate antibiotics for treating dental diseases, and discusses new technology to improve the drug delivery of antibacterial medication by nanotechnology or by 3D printing systems.

## 2. Method

A narrative review where conducted based on several published articles relevant journals and books in Google Scholar PubMed using keywords dental caries, periodontal disease, gingivitis, and related disease we excluded duplicated information and articles that we could not access to full articles.

## 3. Results

Dental caries happens when bacteria convert residual sugars and carbohydrates in the mouth to produce acid that destroys the tooth enamel and its underlying dentin layer.^[21]^ If dental decay is not treated promptly, it can spread from the tooth crown to the root. However, if caries is detected early on, they can be reversed--dental caries resulting from Lactobacilli, Actinomyces, and Streptococcus mutans.^[22]^

Dental caries can be treated by Amoxicillin, metronidazole, co-amoxiclav alone, or clindamycin alone some studies record the use of secnidazole as treatment of dental caries.^[23]^ Gum disease, known as gingivitis, is brought on by plaque accumulation on teeth, which inflames the gums around the teeth. Bacteria are found in plaque naturally. Films that cling to teeth and release chemicals that cause gum irritation. If left untreated, bleeding, puffiness, and redness of the gums can develop into periodontitis.

Actinomyces, prevotella intermedia, streptococcus anginosus, and Campylobacter rectus are possible culprits in gingivitis. Amoxicillin, metronidazole, and tetracyclines (tetracycline, doxycycline, or minocycline) can treat gingivitis.^[24]^

A dangerous gum condition called periodontitis weakens the bone-supporting teeth and affects soft tissue. Plague accumulation results in gradual degradation if left untreated and may lead to tooth loss.^[25]^ Bacteria from periodontitis can enter the bloodstream and damage other body parts if treatment is not received. In cases of periodontal disease, oral bacteria can create ulcerated epithelium that allows the bacteria to enter the circulation. This can lead to temporary bacteremia, considered a concern, particularly in immunocompromised patients or those who wear prosthetics.^[25]^ Porphyromonas gingivitis, bacteroid forsythias, lactobacillus, prevotella intermedia, and fusobcreium nucleatum are a few possible bacteria that cause periodontitis. Oral streptococci can be seen in various extra-oral diseases and has recently been categorized as fastidious and periodontal anaerobes.^[26–28]^ Periodontal infections can be treated by Tetracyclines such as Tetracycline itself, Doxycycline, and Minocycline. prevotella oralis, prevotella melanogenic, streptococcus anginosus, Porphyromonas gingivalis, and Peptostreptococcus micros-caused periapical abscess. Treatment options for periapical abscess include erythromycin, Amoxicillin, and cefoxitin. Acute necrotizing ulcerative gingivitis, sometimes called Vincent angina, is caused by *spirochete borrelia vincentii*, fusiform. It is an acute bacterial infection due to overgrowth of normal oral flora when there are disturbances in oral microbiota. There are 700 bacteria living in the oral cavity as normal flora, most of which are anaerobic.^[29]^ This type of dental infection can be treated by metronidazole or clindamycin or sometimes by penicillins such as penicillin V or penicillin G, Tetracyclines, Amoxicillin, and co-amoxiclav.^[30]^

### 3.1. Pericoronitis

Peptostreptococcus, Porphyromonas gingivalis, and fusobacterium species cause Pericoronitis. It is related to dental cases of the third molar and can be treated with Amoxicillin or metronidazole.

Peri-implantitis is caused by Peptostreptococcus incisors, fusobacteruim nucleatum, prevotella intermedia, pseudomonas aeroginosa, and Staphylococcus species. It can be treated by surgical decontamination, including chemical (use of citric acid, ethylenediaminetetraacetic acid, hydrogen peroxide, saline water), or laser. Surgical treatment provides air power abrasion, respective surgery (implantoplasty), and regenerative surgery. On the other hand, non-surgical include mechanical methods, antiseptic, and topical antibiotics such as tetracycline or doxycycline.^[31]^

Locally administered antibiotics alone or adjunct to surgical and non-surgical treatments for peri-implantitis showed favorable outcomes, albeit with limited evidence. The administration of systemically delivered antibiotics in combination with non-surgical or surgical treatments remained questionable.^[32]^

Endodontic (pulpitis or RC treatment) caused by Peptostreptococcus micros, Porphyromonas endodontalis, prevotella intermedia, prevotella melaninogenica, fusobacterium nucleatum; it can be treated (as non-surgical) by Amoxicillin or clindamycin or Azithromycin.^[33]^

Pulpitis with a periapical abscess is caused by fusarium intermedia, Peptostreptococcus micros, canociytophaga achalasia, Serenoa endodontics, and streptococcus species. It can be treated by Amoxicillin, azithromycin, co-amoxiclav, clarithromycin, Cefoxitin, clindamycin, or metronidazole.^[34]^

Endodontic therapy consists of a series of treatments, including removing pulpal tissue, cleaning, shaping, obturation, and placing a permanent restoration for the tooth.^[7]^

RC treatment includes a sequence for the infected pulp of a tooth intended to result in the elimination of infection and the production of the decontaminated tooth and protection from future microbial invasion. RC and their associated pulp chamber are the physical hollows within a tooth naturally inhabited by nerve tissue, blood vessels, and other cellular entities, causing pain. These dental procedures alleviate pain and prevent future infections of the RC and pulp chamber.^[35]^

Ludwig angina is a bacterial infection (a rare type of cellulitis) that affects the neck and the floor of the mouth.^[36]^ It is not contagious and typically starts from an abscessed tooth; it can spread rapidly, causing life-threatening swelling that can affect the ability to breathe (this is due to edema, which makes breathing difficult).

A dry socket (alveolar osteitis) can happen after dental extraction. When the tooth is removed, a blood clot forms in the socket (a hole in the bone where the tooth was). A dry socket happens when a blood clot moves or does not form. Without the clot, the bone and nerves are exposed to the oral environment, leading to more pain than before extraction. A dry socket can be painful and delay surgical site healing.^[37]^

A dry socket is better, which may be prevented by pre-administration of metronidazole rather than treated by this antimicrobial.^[38]^ Although it is challenging to discover causative bacteria in dry sockets, it has been noted that anaerobic bacteria are primarily responsible for the formation of dry sockets. It has been found that Treponema denticola is known to be a putative microorganism in the development of periodontal disease.^[39]^

It can be treated in the dental office by local antibacterial, antibiotic, antifibrinolytic agents, and steroids; the dentist also uses obtunding dressings such as eugenol-containing. However, oral antibiotics used to treat dry sockets include penicillins (such as penicillin V or penicillin G), clindamycin, metronidazole, and Tetracyclines.

Deep neck space infections, including Para pharyngeal abscess, peritonsillar abscess, and retropharyngeal abscess, commonly arise from an odontogenic or upper aerodigestive tract origin. Both aerobic and anaerobic bacteria may cause this infection.

This condition may be life-threatening if not diagnosed and not treated promptly, as it may lead to airway compromise and spread to adjacent compartments.

Treatment should include broad coverage of beta-lactamase-producing bacteria staphylococcus aureus; other bacteria include streptococcus pyogenes and viridance. Anaerobic gram-negative bacilli and Peptostreptococcus species also take part. Until culture results are obtained to help direct treatment, the empirical treatment includes clindamycin plus levofloxacin as injections because it is a life-threatening condition.^[40]^

For cases with a rhizogenic source of infection, use vancomycin plus ampicillin-sulbactam or vancomycin plus ceftriaxone plus metronidazole or clindamycin plus levofloxacin.

Treatment of jaw osteomyelitis is complicated by the presence of teeth and persistent exposure to the oral environment. Antibiotic therapy tends to be prolonged, often for weeks or months. Clindamycin, Dicloxacillin, and Moxalactam have excellent bioavailability in bone tissue (concentration in the bone about 90% that in the blood). Other antibiotics with good penetrating activity include ciprofloxacin, clindamycin, metronidazole, chloramphenicol, rifampin, and others.

Anaerobic bacteria comprise a significant part of the oral and dental indigenous and pathogenic flora. Anaerobic bacteria in the oral cavity include: Fusobacteria, Bacteroides, eubacteria, propionibacterium, Actinomyces, Peptostreptococcus, Selenomonas, Treponema, prevotella, Porphyromonas^[41]^ These anaerobic bacteria cause many infections such as pulpitis, periodontal, periapical and dental abscesses, perimandubular space infection.

Antibiotics effective in the treatment of anaerobic infections include metronidazole, Meropenem, Co-amoxiclav, pipercillin-clavulanic acid, Ticarcillin-clavulanic acid, amoxicillin-sulbactam, clindamycin, chloramphenicol.^[42]^

Anything that creates a pathway for bacteria to the tooth or surrounding tissues can lead to tooth abscess. Dental caries and chipped or cracked teeth allow bacteria to seep into the path of a tooth and spread to the pulp.^[43]^

Periodontal disease is an infection and inflammation of tissues around the teeth; as the periodontal disease progresses, the bacteria gain access to deeper tissues, causing abscesses. Injury to the teeth, like trauma, can damage the inner pulp even if there is no visible crack; the injury makes it susceptible to infection.^[44]^

A tooth abscess is a pocket of pus from a bacterial infection. An abscess usually looks like a red, swollen bump, boil, or pimple. It affects the involved tooth, but the disease can also spread to the surrounding bone, neighboring teeth, or soft tissue.^[45]^

Dental abscesses can be treated by drainage of pus and using 1 or more of the following antibiotics: Ampicillin-Sulbactam or penicillin G or co-amoxiclav plus metronidazole or Cefoxitin.^[46]^

Peri-implantitis is caused by peptedostreptococcus incisors, fusobacterium nucleatum, prevotella intermedia, pseudomonas aeroginosa, and staphylococcus species. This can be treated by surgical decontamination by laser or chemicals (citric acid, ethylenediaminetetraacetic acid hydrogen peroxide, or saline).

Surgical treatment includes air power abrasion, respective surgery (implantoplasty), and regenerative surgery. Non-surgical procedures include mechanical methods, antiseptic, and topical antibiotics such as tetracycline or doxycycline^[47]^ all the above summarized in Table 1.

**Table 1:** Type of infection and treatment agent.

Type of infection	Causative microorganism	First-choice antibiotic	Second and third-choice antibiotics
Dental caries (after periodontal work)	Streptococcus mutans, Actinomyces species, lactobacilli.	Metronidazole + Amoxicillin or Co-Amoxiclav	Clindamycin
Gingivitis	Campylobacter rectus, Actinomyces, prevotella intermedia, streptococcus anginosus	Tetracyclines (tetracycline doxycycline or minocycline)	Amoxicillin or metronidazole
Periodontitis	Porphyromonas gingivalis, bacteroid forsythias, Actinobacillus, prevotella intermedia, fusobacterium nucleatum	Tetracycline	Doxycycline or minocycline
Periapical abscess	Peptostreptococcus micros, prevotella oralis, prevotella melanogenic, streptococcus anginosus, Porphyromonas gingivalis	Amoxicillin or Azithromycin	Cefoxitin
Acute necrotizing ulcerative gingivitis or Vincent angina	Spirochete Borrelia vincent, and gram-negative bacillus fusiformis fusiform	Penicillin or metronidazole	Clindamycin
Pericoronitis	Peptostreptococcus micros, Porphyromonas gingivalis, fusobacter species	Metronidazole	Amoxicillin
Peri-implantitis	Peptostreptococcus intros, fusobacterium nucleatum, prevotella intermedia, pseudomonas aeroginosa, staphylococcus species	Non-surgical include amoxicillin or metronidazole or tetracycline	Ciprofloxacin
Endodontists	Peptostreptococcus micros, Porphyromonas endodontics, prevotella intermedia, prevotella melanogenic, fusobacterium nucleatum	Amoxicillin	Clindamycin
Pulpitis with periapical dental abscess	Fusobacterium nucleatum, prevotella intermedia, Peptostreptococcus micros, capnociytophaga achalasia, Serenoa endodontics	Amoxicillin or Azithromycin	Cefoxitin
Cellulitis	Staphylococcus methicillin-resistant staphylococcus aureus, beta-hemolytic streptococcus group A, streptococcus pyogenes.One, Hemophilus species, and anaerobic bacteria such as fusobacteria, prevotella, Porphyromonas	Penicillin Cephalexin Dicloxacillin Ceftriaxone Cephazolin	Clindamycin
Dry socket	Treponema denticola	Metronidazole	Azithromycin
Infected socket	Due to the exposure to the jawbone, there is no involvement of bacteria	No antibiotic cleaning and analgesic	No antibiotic but metronidazole for prevention
Dental abscess	Bacteroides, fusobacterium, Actinomyces, Peptostreptococcus, Peptococcus, Porphyromonas, prevotella oralis, and melanogenic	Ampicillin-sulbactam, amoxiclav. Penicillin G plus metronidazole or Cefoxitin	Clindamycin plus metronidazole
Ludwig angina	The disease is usually polymicrobial, involving oral flora, both aerobes and anaerobes. The most common organisms are streptococcus, staphylococcus,peptostreptococcus, fusobacterium, Bacteroides, and Actinomyces	Ampicillin-sulbactam or clindamycin	Add Cefepime Meropenem or piperacillin-tazobactam to cover pseudomonas aeroginosa in immunosuppressed patients.
Osteomyelitis on the face	The prime opportunistic pathogenic species are streptococci and anaerobic bacteria. hematogenous osteomyelitis, there are staphylococci; enteric rods are the most specific bacteria isolated from the blood or bone	Clindamycin (given IV in first 1–2 wk) Rifampin Trimethoprim-sulfamethoxazole	Moxifloxacin
Deep head and neck space infection	Staphylococcus aureus, streptococcus pyogenes. One, streptococcus variance, anaerobic gram-negative bacilli, Peptostreptococcus	Parenteral clindamycin plus parenteral levofloxacin	–
Anaerobic infections	Fusobacterium, Bacteroides, propionibacterium, Actinomyces, Peptostreptococcus, selenomonas, treponema, prevotella, Porphyromonas	Metronidazole, Meropenem, Amoxiclav, Piperacillin-clavulanic acid, Ticarcillin-clavulanic acid, amoxicillin-sulbactam, clindamycin, chloramphenicol, Cefoxitin	Clindamycin plus metronidazole

### 3.2. The uses of nanotechnology and biomaterials for dental implant medication delivery

Nanostructured scaffolds and matrices aid in better controlling cell differentiation in regenerative dentistry. Compared to traditional autologous and allogenic tissues or alloplastic materials, nanomaterials better mimic the natural architecture and structure of teeth and generate functional tissues. Furthermore, some metals are used in medication complexes to improve transportation. Different methodologies are offered for biomaterial-based delivery systems, despite the fact that each biomaterial has pros and cons that can greatly impact complicated transportation, such as solubility in physiological settings or dispersion in tissues. Biomaterials can prolong time contact, have antibacterial and anti-inflammatory properties, and even strengthen the effects of antibiotics for treating oral infections. Furthermore, these biomaterials are frequently made into injectable systems, gels, hydrogels, microspheres, fibers, and particulate complexes.^[48–51]^

### 3.3. 3D printing designs unique drug delivery systems

The uses of 3D printing in medical devices, films, liquids, gastroretentive, colon, transdermal, and intrauterine drug delivery systems. Owing to its unique characteristics and originality, 3D printing is inherently capable of resolving several formulation and drug delivery issues, many of which are connected to medications that are poorly soluble in water. The recent FDA technical advice on additive manufacturing in relation to medical devices and the approval of Spritam®, Laboratory Prasco S.P A have sparked a great deal of research in the fields of bioengineering and drug delivery systems. From the pre-clinical stage to first-in-human trials and on-site manufacture of bespoke formulations with exceptional dosage flexibility at the point of treatment, 3D printing technology might be effectively applied. The pharmaceutical manufacturing industry will undergo a quick transition with the adoption of new regulatory rules and the advent of breakthrough 3D printing machines that provide built-in quality and flexibility.^[52]^

## 4. Discussion

Despite the desire to reduce antibiotics in dental infections, antibiotics are effective in the treatment of the following dental infections: acute necrotizing ulcerative gingivitis, stage III grade C/incisor-molar pattern periodontitis, acute periapical abscess, cellulitis, local or systemic spread infection, pericoronitis, peri-implantitis, deep facial layers of the head and neck infection, Ludwig angina, anaerobic infections.^[53]^

Amoxicillin is a broad-spectrum penicillin that is destroyed by bacterial penicillin. It treats dental caries, gingivitis, periapical abscesses, pericoronitis, endodontitis, and pulpitis with apical abscesses. Amoxicillin is a first-line antibiotic in dental infections and a frequently prescribed antibiotic in dental practice (approximately 50% of prescriptions). Some dentists prescribe metronidazole with Amoxicillin to cover most likely pathogens, particularly anaerobic microorganisms, which usually inhabit the oral cavity.^[54]^

Other dentists prefer using co-amoxiclav to broaden the spectrum to include penicillinase-producing bacteria. This preparation combines Amoxicillin and clavulanic acid. Clavulanic acid is β-lactamase inhibitor. The disadvantages of clavulanic acid are hepatotoxicity and that it is better to be avoided during pregnancy. It has been shown that all the bacteria extracted from odontogenic abscesses were susceptible to co-amoxiclav.^[50]^

Co-amoxiclav is helpful in the treatment of dental caries, dental abscesses, and anaerobic infections. Other penicillins, such as penicillin V and penicillin G, were used in the treatment of Vincent angina, cellulitis, and abscesses. Ampicillin-sulbactam is used to treat dental abscesses and Ludwig angina. Meropenem plus piperacillin-tazobactam when an infection is caused by nasty microorganisms such as Pseudomonas aeroginosa and Staphylococcus aureus.^[51]^

The third most commonly used antibiotic is metronidazole, which is primarily effective against anaerobic bacteria and used in the treatment of dental caries (the causative microorganism is streptococcus mutans), gingivitis, and acute necrotizing ulcerative gingivitis, pericoronitis, and dental abscess, dry or infected socket.^[52]^

Clindamycin has 2 good characteristics. First, it is effective against most anaerobic infections and is a good bone penetrator. However, the most severe and dangerous side effect of clindamycin is the overgrowth of clostridium difficile bacteria, causing pseudomembranous colitis, a dangerous complication.

Unfortunately, there is antagonism between clindamycin and metronidazole as only 1 study was done by researchers,^[53]^ but most researchers, such as,^[53]^ used clindamycin with metronidazole to prevent dry socket and pseudomembranous colitis at the same time (prevent the side effect).

Clindamycin is helpful in the treatment of dental caries, Vincent angina, endodontitis, cellulitis, jaw osteomyelitis, dental abscess, Ludwig angina, and anaerobic infections. It has been found that clindamycin is as effective as amoxicillin and metronidazole in treating periodontitis in diabetic patients.^[55]^

Doxycycline (a type of tetracycline) is used in the treatment of gingivitis and periodontitis, and minocycline is also a type of tetracycline used topically during surgical work in the treatment of peri-implantitis.

Dentists have prescribed many Cephalosporins to treat dental infections in penicillin-allergic patients if the patient has not had severe reactions to Cephalosporins.^[56]^ However, it is well known that there is a 10% cross-allergenicity between penicillins and Cephalosporins, which necessitates avoiding Cephalosporins to treat infections in patients allergic to penicillin, and it is better to use Azithromycin, particularly in the treatment of periapical abscess, dental abscess, dry socket.^[57]^

Cefoxitin is used to treat some dental infections when anaerobic bacteria are suspected. Other Cephalosporins are also used in dentistry, such as cephalexin, Cephazolin, and ceftriaxone. Cefepime is needed when the presence of pseudomonas aeroginosa is suspected.

Fluoroquinolones are used in different dental diseases; for example, ciprofloxacin is used in treating peri-implantitis, and moxifloxacin is used in treating jaw osteomyelitis. Levofloxacin is indicated in the treatment of deep head and space infection. Some other antibiotics, such as rifampin, have little place in dentistry; it is used in the treatment of osteomyelitis of the face. Several studies link some types of cancer and bacterial infection,^[58,59]^ there is association between oral cancer and the infections for that reason our review is important to surmise all these factors and related illness.

## 5. Conclusions

The most commonly used antibiotic in dental infections is Amoxicillin (approximately 50%); the second most commonly used antibiotic to treat dental infections is co-amoxiclav. Cephalosporins are better to avoid in penicillin-allergic patients because there is 10% cross-allergenicity between penicillins and cephalosporins due to the similarity in the primary structural formula (beta-lactam ring).

It has been found that combining Amoxicillin and metronidazole is effective in stage I to III grade c periodontitis. Also, this combination is effective in the treatment of aggressive periodontitis.

The second combination found to be effective in dental infections is co-amoxiclav and metronidazole.

On the other hand, the combination of clindamycin and metronidazole shows some antagonism, but it could be used in some impotent situations to prevent the dangerous pseudomembranous colitis resulting from the use of clindamycin. Clindamycin inhibits most intestinal flora’s growth, and the resistant clostridium difficle overgrows. These bacteria secrete toxins that cause narcotization to intestinal cells, leading to pseudomembranous colitis.

For empirical use of antibiotics in most dental infections, a combination of clindamycin and metronidazole can be used in penicillin-allergic patients. Most previous work agrees with this use, except a report from some research indicates the presence of drug resistance to this combination that decreases the effect. Besides the normal methods, emerging technologies such as 3D printing for drug delivery of antibiotics and disinfectants hold promise in enhancing treatment efficacy and patient outcomes. By leveraging the precision and customization afforded by 3D printing, dentistry can tailor antimicrobial interventions to individual patient needs, optimizing therapeutic outcomes while minimizing adverse effects. Furthermore, the integration of nanotechnology and biomaterials into dental implant medication delivery represents a paradigm shift in preventive and therapeutic approaches. Nanotechnology enables the development of novel drug delivery systems with enhanced bioavailability and targeted delivery, potentially reducing the risk of infection and improving implant success rates. Moreover, biomaterials tailored for controlled release mechanisms offer prolonged and localized antimicrobial activity, fostering tissue regeneration and implant integration.

Clindamycin acts on bacterial protein synthesis, and metronidazole acts on bacterial nucleic acid synthesis; this needs to be clarified in future clinical trials in dentistry.

## Acknowledgments

We thank the Iraqi Medical Research Center for their support and providing us with the research materials.

## Author contributions

**Conceptualization:** Thamir Hani Alsafar.

**Data curation:** Thamir Hani Alsafar.

**Formal analysis:** Thamir Hani Alsafar, Tahani Abdulaziz Alsandook, Faraj Mohammed Abdullah, Hany A. Al-Hussaniy.

**Funding acquisition:** Tahani Abdulaziz Alsandook.

**Investigation:** Faraj Mohammed Abdullah.

**Methodology:** Tahani Abdulaziz Alsandook, Faraj Mohammed Abdullah.

**Project administration:** Faraj Mohammed Abdullah, Amjad I Oraibi.

**Resources:** Hany A. Al-Hussaniy, Amjad I Oraibi.

**Software:** Amjad I Oraibi.

**Supervision:** Tahani Abdulaziz Alsandook.

**Writing – original draft:** Thamir Hani Alsafar, Qais Y. Hatim.

**Writing – review & editing:** Wael Lutfi, Qais Y. Hatim, Hany A. Al-Hussaniy.

## References

[1] Martínez M Postolache TT García-Bueno B. The role of the oral microbiota related to periodontal diseases in anxiety, mood and trauma-and stress-related disorders. Front Psychiatry. 2022;12:814177. 35153869 10.3389/fpsyt.2021.814177 PMC8833739

[2] Al-Hussaniy HA Alburghaif AH Naji MA. Leptin hormone and its effectiveness in reproduction, metabolism, immunity, diabetes, hopes and ambitions. J Med Life. 2021;14:600–5. 35027962 10.25122/jml-2021-0153 PMC8742898

[3] Sabharwal A Stellrecht E Scannapieco FA. Associations between dental caries and systemic diseases: a scoping review. BMC Oral Health. 2021;21:472. 34563194 10.1186/s12903-021-01803-w PMC8466895

[4] Abdulameer AA Mohammed ZN Tawfeeq KT. Endoscopic characteristics and management of subepithelial lesions in VideoGastascopie. Med Pharm J. 2022;1:4–13.

[5] Marah ZAA Abdulkareem AA Gul SS Alshami ML. A survey of systemic antibiotic prescription patterns amongst Iraqi dentists. Int Dent J. 2022;72:338–45. 34344542 10.1016/j.identj.2021.06.002 PMC9275136

[6] Awad MM Elsahar MI. Asymptomatic bacterial infection in pregnancy: a new update. Med Pharm J. 2022;1:1–11.

[7] Kula L Kalinowska J Koczor-Rozmus A. Endodontic treatment regimens and their application in practice-survey and comparative study. J Educ Health Sport. 2021;11:30–43.

[8] Zieniewska I Maciejczyk M Zalewska A. The effect of selected dental materials used in conservative dentistry, endodontics, surgery, and orthodontics as well as during the periodontal treatment on the redox balance in the oral cavity. Int J Mol Sci. 2020;21:9684. 33353105 10.3390/ijms21249684 PMC7767252

[9] Rusu A Tiliscan C Adamescu AI. Carbapenemase-producing uropathogens in real life: epidemiology and treatment at a County Emergency Hospital from Eastern Romania. J Med Life. 2023;16:707–11. 37520479 10.25122/jml-2023-0139 PMC10375344

[10] Aldafaai RR Jafar Z Al-Rubbaey Y. Impact of dental anxiety on dental caries and salivary alkaline phosphatase in children across different nutritional statuses. J Med Life. 2023;16:1540–5. 38313167 10.25122/jml-2023-0085 PMC10835552

[11] Cherepyuk O Oktysyuk Y Bazalytska A Rozhko M. Correction of disordered oral immunity in children affected by dental caries with herbal immune modulator “Esberitox.”. Pharmacia. 2020;67:347–50.

[12] Maan LA Lachguer FZ Bouziane A. Infectious healthcare waste management among private dental practitioners in the Rabat-Salé-Kénitra region, Morocco: a cross-sectional study on knowledge, attitudes, and practices. Front Cell Infect Microbiol. 2023;16:1084–92. 10.25122/jml-2023-0038 PMC10600657 37900083

[13] Ahmadi H Ebrahimi A Ahmadi F. Antibiotic therapy in dentistry. Int J Dent. 2021;2021:6667624. 33574843 10.1155/2021/6667624 PMC7861949

[14] Thornhill MH Gibson TB Durkin MJ. Prescribing of antibiotic prophylaxis to prevent infective endocarditis. J Am Dent Assoc. 2020;151:835–45.e31. 33121605 10.1016/j.adaj.2020.07.021 PMC7959887

[15] Hadi HM Shahada AR Hussein NM. Doxorubicin side effects and its uses a new update: a narrative review. Arabian J Drug Res. 1923;1:1–6.

[16] Hamad AJ Albdairi AJ Alkemawy SN. Assessment of the incidence and etiology of nosocomial diarrhea in a medical ward in Iraq. Antibiotics (Basel). 2022;15:132–7. 10.25122/jml-2021-0275 PMC8852629 35186147

[17] Ali A Bhosale A Pawar S Kakti A Bichpuriya A Agwan MA. Current trends in root canal irrigation. Cureus. 2022;14:e24833. 35698671 10.7759/cureus.24833 PMC9184175

[18] Rathod A Jaiswal P Bajaj P Kale B Masurkar D. Implementation of low-level laser therapy in dentistry: a review. Cureus. 2022;14:e28799. 36225465 10.7759/cureus.28799 PMC9534528

[19] Juarranz A Gilaberte Y González S. Photodynamic therapy (PDT) in oncology. Cancers (Basel). 2020;12:3341. 33198063 10.3390/cancers12113341 PMC7698223

[20] Haki M Haeder A Al-Tameemi Z. Review of multiple sclerosis: epidemiology, etiology, pathophysiology, and treatment. Medicine (Baltimore). 2024;103:e37297. 38394496 10.1097/MD.0000000000037297 PMC10883637

[21] Rowińska I Szyperska-Ślaska A Zariczny P Pasławski R Kramkowski K Kowalczyk P. The influence of diet on oxidative stress and inflammation induced by bacterial biofilms in the human oral cavity. Materials (Basel). 2021;14:1444. 33809616 10.3390/ma14061444 PMC8001659

[22] de Oliveira RVD Bonafé FSS Spolidorio DMP. Streptococcus mutans and Actinomyces naeslundii interaction in dual-species biofilm. Microorganisms. 2020;8:194. 32023892 10.3390/microorganisms8020194 PMC7074783

[23] Raheema DA Kassab HJ. Preparation and in-vitro evaluation of secnidazole as periodontal in-situ gel for treatment of periodontal disease. Iraqi J Pharm Sci. 2022;31:50–61.

[24] Castañeda-Corzo G-J Infante-Rodríguez L-F Villamil-Poveda J-C Bustillo J Cid-Arregui A García-Robayo D-A. Association of Prevotella intermedia with oropharyngeal cancer: a patient-control study. Heliyon. 2023;9:e14293. 36938439 10.1016/j.heliyon.2023.e14293 PMC10018557

[25] Elbelkemy MF Mostafa RW Ibrahim RO. Periodontal health knowledge among patients with fixed orthodontic appliance: a hospital based cross-sectional study. Adv Dent J. 2024;6:155–74.

[26] Shaaban SM Gaber Z Semary S Dewidar AM. Impact of vitamin B12 on outcome of early stage luminal A and B breast cancer, single center experience. Med Pharm J. 2023;2:17–27.

[27] Al-Kelaby WJ Kaabi A Alhussaniy ZS. Histological and histochemical studies of the eye structure of Anas platyrhynchus (Mallard) duck species. Indian Vet J. 2023;100:7–15.

[28] Al-Hussaniy H Al-Tameemi Z Al-Zubaidi B. Pharmacological properties of Spirulina species: hepatoprotective, antioxidant and anticancer effects. Farmacia. 2023;71:670–8.

[29] Mehdipour A Ehsani A Samadi N. The antimicrobial and antibiofilm effects of three herbal extracts on compared with chlorhexidine 0.2% (study). Biology (Basel). 2022;15:526–32. 10.25122/jml-2021-0189 PMC9126453 35646170

[30] Veeraraghavan B Bakthavatchalam YD Sahni RD. Orally administered amoxicillin/clavulanate: current role in outpatient therapy. Infect Dis Ther. 2021;10:15–25. 33306184 10.1007/s40121-020-00374-7 PMC7954971

[31] Rokaya D Srimaneepong V Wisitrasameewon W Humagain M Thunyakitpisal P. Peri-implantitis update: risk indicators, diagnosis, and treatment. Eur J Dent. 2020;14:672–82. 32882741 10.1055/s-0040-1715779 PMC7536094

[32] Pinto KP Barbosa AFA Silva EJNL Santos APP Sassone LM. What is the microbial profile in persistent endodontic infections? A scoping review. J Endod. 2023;49:786–98.e7. 37211309 10.1016/j.joen.2023.05.010

[33] Zhou S He TC Zhang Y Zhang H. Comparison of the main pathogenic microorganisms of various common oral diseases in children and adults. Pediatr Discov. 2023;1:e35. 38371743 10.1002/pdi3.35 PMC10874635

[34] Gulabivala K Ng YL. Factors that affect the outcomes of root canal treatment and retreatment-A reframing of the principles. Int Endod J. 2023;56(Suppl 2):82–115. 36710532 10.1111/iej.13897

[35] Alpakistany T Taha TM Gazi KS Thabet MA Hroobi AA Melebari M. β-lactamases-dependent antimicrobial resistance in enterobacteria isolated from commercial poultry farms in the Makkah province, Saudi Arabia. J App Biol Biotech. 2024;12:231–8.

[36] Kamal A Salman B Abdul Razak NH Qabbani AA Samsudin AR. The efficacy of concentrated growth factor in the healing of alveolar osteitis: a clinical study. Int J Dent. 2020;2020:9038629. 32454827 10.1155/2020/9038629 PMC7240629

[37] Ali AH Karabeit Z Nesser A. Comparison between the efficiency of aloevera extract and alvogyl in dry socket (alveolar osteitis) management. Int J Dentistry Oral Sci. 2021;8:1578–82.

[38] Al-Hussaniy H Altalebi RR Albu-Rghaif AH. The use of PCR for respiratory virus detection on the diagnosis and treatment decision of respiratory tract infections in Iraq. J Pure Appl Microbiol. 2022;16:201–6.

[39] Paul R Chaudhury N Chattopadhyay S. Bacteriological profile of anaerobes in deep-seated abscess of patients attending a tertiary care hospital. Asian J Med Sci. 2022;13:149–53.

[40] Hampelska K Jaworska MM Babalska ZL Karpiński TM. The role of oral microbiota in intra-oral halitosis. J Clin Med. 2020;9:2484. 32748883 10.3390/jcm9082484 PMC7465478

[41] Mahmoud FM Khedr RA Ebeid E El-Mahallawy HA Hassan SS. Impact of rapid molecular diagnostic technique on time to optimal antimicrobial therapy and hospital outcomes in pediatric cancer patients with sepsis. Asian Pac J Cancer Prev. 2023;24:2465–71. 37505781 10.31557/APJCP.2023.24.7.2465 PMC10676468

[42] Arhun N Arman-Özçirpici A Çehreli SB. The restorative dentist and orthodontist: orthodontic implications of dental caries, tooth fracture, exposed dental pulp, and esthetic improvements. Integr Clin Orthod. 2023:345–410.

[43] Martínez-García M Hernández-Lemus E. Periodontal inflammation and systemic diseases: an overview. Front Physiol. 2021;12:709438. 34776994 10.3389/fphys.2021.709438 PMC8578868

[44] Nautiyal A Bali S Pradeepkumar V. Dental Abscess A diagnostic enigma. J Pharm Negat Results. 2022;239:239–43.

[45] Beshna E Ashour RA Layas NA. Pharmacovigilance knowledge, attitudes, and practices of pharmacists in Zawia (Libya). Med Pharm J. 2023;2:99–106.

[46] Hakim LK Yari A Nikparto N. The current applications of nano and biomaterials in drug delivery of dental implant. BMC Oral Health. 2024;24:126. 38267933 10.1186/s12903-024-03911-9 PMC10809618

[47] Altalebi RR Al-Hussaniy HA Al-Tameemi ZS. Non-alcoholic fatty liver disease: relation to juvenile obesity, lipid profile, and hepatic enzymes. J Med Life. 2023;16:42–7. 36873135 10.25122/jml-2022-0091 PMC9979179

[48] Tebyaniyan H Hussain A Vivian M. Current antibacterial agents in dental bonding systems: a comprehensive overview. Future Microbiol. 2023;18:825–44. 37668450 10.2217/fmb-2022-0203

[49] Tahmasebi E Keshvad A Alam M. Current infections of the orofacial region: treatment, diagnosis, and epidemiology. Life (Basel). 2023;13:269. 36836626 10.3390/life13020269 PMC9966653

[50] Mosaddad SA Hussain A Tebyaniyan H. Green alternatives as antimicrobial agents in mitigating periodontal diseases: a narrative review. Microorganisms. 2023;11:1269. 37317243 10.3390/microorganisms11051269 PMC10220622

[51] Yazdanian M Rahmani A Tahmasebi E Tebyanian H Yazdanian A Mosaddad SA. Current and advanced nanomaterials in dentistry as regeneration agents: an update. Mini Rev Med Chem. 2021;21:899–918. 33234102 10.2174/1389557520666201124143449

[52] Jacob S Nair AB Patel V Shah J. 3D printing technologies: recent development and emerging applications in various drug delivery systems. AAPS PharmSciTech. 2020;21:220. 32748243 10.1208/s12249-020-01771-4

[53] Kilmukhametova YH Batig VM Ostafiichuk MA. Indicators of antioxidant protection of blood in necrotizing ulcerative gingivitis in experimental animals. J Med Life. 2021;14:68–74. 33767788 10.25122/jml-2020-0149 PMC7982257

[54] Abass AA Al-Magsoosi MJ Kadhim WA. Antimicrobial effect of Red Roselle (Hibiscus Sabdariffa) against different types of oral bacteria. J Med Life. 2022;15:89–97. 35186141 10.25122/jml-2021-0184 PMC8852637

[55] Krishna KV Koujalagi K Surya RU Namratha MP Malaviya A. Enterococcus species and their probiotic potential: current status and future prospects. J App Biol Biotech. 2022;11:36–44.

[56] Mili C Kalita S Roy S. Microbes as a potential bioremediation tool for atrazine-contaminated soil: a review. J App Biol Biotech. 2022;11:8–15.

[57] Mohiuddin M Iqbal Z Farooq M. Multidrug resistance in clostridia.Faisalabad, Pakistan: Animal Health Perspectives, Unique Scientific Publishers. 2022;2:34–43.

[58] Suárez-Fernández C Barrientos C García-Pola M. Public awareness on oral cancer: a population-based study in Asturias. Asian Pac J Cancer Prev. 2023;24:4127–31. 38156847 10.31557/APJCP.2023.24.12.4127 PMC10909085

[59] Zaib N Maqsood A Ghayas S Ansari F Kiyani A Masood R. Analysis of discrepancy index between clinical and histopathological diagnosis of oral lesions. Asian Pac J Cancer Prev. 2023;24:3207. 37774073 10.31557/APJCP.2023.24.9.3207 PMC10762748

